# Increased flux in acetyl-CoA synthetic pathway and TCA cycle of *Kluyveromyces marxianus* under respiratory conditions

**DOI:** 10.1038/s41598-019-41863-1

**Published:** 2019-03-29

**Authors:** Yuri Sakihama, Ryota Hidese, Tomohisa Hasunuma, Akihiko Kondo

**Affiliations:** 1 Graduate School of Innovation, Science and Technology, Kobe University, 1-1 Rokkodai, Nada, Kobe, 657-8501 Japan; 2 RIKEN Center for Sustainable Resource Science, 1-7-22 Suehiro, Tsurumi, Yokohama, Kanagawa 230-0045 Japan

## Abstract

Yeasts are extremely useful, not only for fermentation but also for a wide spectrum of fuel and chemical productions. We analyzed the overall metabolic turnover and transcript dynamics in glycolysis and the TCA cycle, revealing the difference in adaptive pyruvate metabolic response between a Crabtree-negative species, *Kluyveromyces marxianus*, and a Crabtree-positive species, *Saccharomyces cerevisiae*, during aerobic growth. Pyruvate metabolism was inclined toward ethanol production under aerobic conditions in *S*. *cerevisiae*, while increased transcript abundances of the genes involved in ethanol metabolism and those encoding pyruvate dehydrogenase were seen in *K*. *marxianus*, indicating the augmentation of acetyl-CoA synthesis. Furthermore, different metabolic turnover in the TCA cycle was observed in the two species: malate and fumarate production in *S*. *cerevisiae* was higher than in *K*. *marxianus*, irrespective of aeration; however, fluxes of both the reductive and oxidative TCA cycles were enhanced in *K*. *marxianus* by aeration, implying both the cycles contribute to efficient electron flux without producing ethanol. Additionally, decreased hexokinase activity under aerobic conditions is expected to be important for maintenance of suitable carbon flux. These findings demonstrate differences in the key metabolic trait of yeasts employing respiration or fermentation, and provide important insight into the metabolic engineering of yeasts.

## Introduction

In the context of bio-refining, microbial fermentation of biomass has been shown to yield a wide spectrum of fuels and chemicals. The commonly used yeast *Saccharomyces cerevisiae* is useful in the field of metabolic engineering because the micro-organism is well-characterized in terms of physiology, biochemistry, and genetics, and an advanced genetic toolbox is available^1–3^. However, *S*. *cerevisiae*’s inherent high ethanol production ability under aerobic conditions (which defines the species as “Crabtree-positive^4–6^”) results in the low yield of other metabolites such as organic acids, diols, and diamines. Therefore, this property often represents a hurdle in the application of this yeast toward bio-refining^7^. In *S*. *cerevisiae*, NADH is oxidized in glucose-rich media by fermentation rather than by respiration, even in the presence of dissolved oxygen^8^. This tendency is attributed to a limited capacity of respiratory dissimilation of pyruvate^4^. Recently, a Crabtree-negative *S*. *cerevisiae* was developed through rational engineering of pyruvate metabolism^9^.

*Kluyveromyces marxianus*, a non-conventional thermo-tolerant yeast, is inherently Crabtree-negative, showing no ethanol production ability after a glucose pulse is provided to respiring cells^10^. In addition, *K*. *marxianus* is distinct among the yeast species in that it can assimilate a variety of sugars, not only glucose but also arabinose, cellobiose, lactose, and xylose. Thus, the species has the desirable properties of being able to produce commodity chemicals from a feedstock of lignocellulosic materials^11–13^. Several genetic tools for *K*. *marxianus* recently have been developed: heterologous gene expression is available by a plasmid-based system^14^ or by integrating into the genome by homologous recombination^15^; efficient non-homologous end-joining activity that joins DNA ends in a sequence-independent manner through transformation has been reported^16^; and the complete genome sequence of *K*. *marxianus* was revealed^17^. Hence, *K*. *marxianus* has attracted considerable attention as a host strain for the production of bio-based chemicals^18,19^. It is known that *K*. *marxianus* grows well, replicating faster under aerobic conditions without ethanol fermentation; however, *K*. *marxianus*’s overall metabolic turnover in glycolysis and the tricarboxylic acid (TCA) cycle and dynamics of the corresponding gene transcripts remain unclear.

A multi-omics analysis allows kinetic visualization of carbon metabolism and identification of the limiting steps in central metabolic pathways such as glycolysis and the TCA cycle, facilitating our understanding of cellular behaviors and molecular networks through different types of time-series data. In the present study, the transcript levels of the genes involved in glycolysis and the TCA cycle and the pool sizes of metabolites were investigated through global qPCR and metabolome analyses to unveil dynamic changes in transcript abundance and intracellular metabolites, respectively, by *K*. *marxianus* and *S*. *cerevisiae* growing with aeration. Furthermore, dynamic metabolic profiling, which directly measures the turnover of metabolic intermediates via *in vivo* ^13^C-labeling of metabolites^20–22^, successfully demonstrated the distinct metabolic responses of Crabtree-positive and –negative yeasts to aeration. Our results indicated that *K*. *marxianus* strictly controls central metabolism, including glycolysis and the TCA cycle, in response to aeration, discriminating between fermentation and respiration.

## Materials and Methods

### Aerobic and anaerobic cultivation of yeast

*S*. *cerevisiae* BY4741 (genotype: *MAT*a *his3Δ1 leu2Δ0 met15Δ0 ura3Δ0*) and *K*. *marxianus* NBRC 1777^17^ were pre-cultivated at 30 °C in YPD medium (20 g/L peptone, 10 g/L yeast extract, 20 g/L glucose) for 16 h under aerobic conditions. The grown cells were collected by centrifugation at 3000 × *g* for 10 min at 4 °C, washed with sterile distilled water, and then inoculated into a 100 mL of YPD broth containing 91 mL of YP base (10 g/L yeast extract and 20 g/L peptone) and 5.5 g/L glucose in a BJR-25NAIS-8M (ABLE Co., Tokyo, Japan) mini fermenter. Glucose (500 g/L) was continuously fed into 100 mL of the medium at 1 mL/h for 9 h of cultivation, yielding a final added amount of 5 g glucose to the culture. Aerobic cultivation was conducted at an aeration rate of 1.0-vvm at 30 °C. The dissolved oxygen concentration was maintained at a level of ≥2.25 ppm by controlling agitation speed. Anaerobic cultivation (fermentation) was performed under the same conditions but without agitation. For all cultures, the initial cell concentration was adjusted to 12.5 g of wet cells/L. Wet cell weight was determined by weighing a cell pellet harvested by centrifugation at 3,000 × *g* for 5 min. Dry cell weight (DCW) was determined by fitting the optical density at 600 nm (OD_600_; monitored using a UVmini-1240 spectrophotometer (Shimadzu, Kyoto, Japan)) to an equation based on a linear correlation between DCW and OD_600_.

For quantification of ethanol and glucose in the fermentation medium, the culture was centrifuged at 3,000 × g at 4 °C for 10 min, and the resulting supernatant was analyzed using an electrochemical biosensor system BF-7 (Oji Scientific Instruments, Hyogo, Japan) operated at 30 °C, with phosphate buffer (pH 7.0) at a flow rate of 1.0 mL/min. Acetate in the fermentation medium was monitored by a high-performance liquid chromatography (HPLC) system (Shimadzu) equipped with an Aminex HPX-87H column (7.8 × 300 mm; Bio-Rad, Hercules, CA) and a refractive index detector (RID-10A; Shimadzu). The HPLC system was operated at 50 °C using 5 mM H_2_SO_4_ as the mobile phase at a flow rate of 0.6 mL/min.

### Pool size analysis of intracellular metabolites

Culture medium (5 mL) was mixed with 7 mL of methanol pre-cooled to −40 °C. The mixture was immediately placed back in the cryostat (−40 °C). After centrifugation at 5,000 × *g* for 5 min at −20 °C, the resulting pellet was suspended by addition of 10 μL of 17 μM camphorsulfonic acid as an internal standard for liquid chromatography–triple quadrupole mass spectrometry (LC-QqQ-MS) analysis; intracellular metabolites were prepared by the boiling ethanol extraction method as described previously^1^. The extract was dried in a vacuum environment using a CVE-3100 centrifugal evaporator (EYELE, Tokyo, Japan), dissolved in 50 μL of Milli-Q water, and applied to LC-QqQ-MS. The LC-QqQ-MS system (LC: Agilent 1200 series; MS, Agilent 6460 Agilent Technologies, Palo Alto, CA, USA) was controlled with a Mastro C18 column (Shimadzu GLC, Ltd.; 150 × 2.0 mm; particle size, 3 μm) as described previously^23^. The pool sizes of target metabolites were calculated based on the area of peaks identified by comparing the chromatographic characteristics of samples with those of authentic standards using MassHunter Quantitative Analysis ver. B04.00 software (Agilent Technologies). Each pool size was represented as µmol of metabolite/g-dry cell weight (µmol/g-DCW).

### qPCR

Each culture of cells was grown for 4 h and total RNA was extracted using a Total RNA Isolation Mini Kit (Agilent Technologies). The concentration of total RNA was determined using a Nano Vue Plus spectrophotometer (Life Technologies, Carlsbad, CA). The quality was checked using an Agilent 2100 Bioanalyzer (Agilent Technologies). cDNA was generated from the extracted total RNA by reverse transcription using ReverTra Ace qPCR RT Mix and gDNA Remover (Toyobo, Osaka, Japan). Quantitative PCR experiments were carried out in triplicate using Thunderbird SYBR qPCR Mix (Toyobo) and an Mx3005P Real Time PCR system (Agilent Technologies). Specific primers for the amplification of the selected genes and *ACT1* as a house-keeping internal-standard gene are listed in Supplementary Table 1. The fold-change of the transcript levels was calculated using the 2^−∆∆CT^ method^24^.

### ^13^C-labeling metabolomics

^13^C-labeling was initiated by adding 1 mL of 500 g/L [U-^13^C] glucose to the 100 mL of YPD broth after 4 h of cultivation. Culture samples were collected at the indicated time points (10 sec, 30 sec, and 1, 2, 5, and 10 min) after the addition of [U-^13^C] glucose; each intracellular metabolite was prepared as described above (in “Pool size analysis of intracellular metabolites”). Extracted metabolites were analyzed using capillary electrophoresis-mass spectrometry (CE-MS) (CE, Agilent G7100; MS, Agilent G6224AA LC/MSD TOF; Agilent Technologies). Mass spectral peaks were identified by searching for mass shifts between the ^12^C- to ^13^C-mass spectra. The ratio of ^13^C to total carbon, defined as the ^13^C fraction, was calculated by relative isotopomer abundance of metabolites incorporating ^13^C atoms as described previously^22^. The relative isotopomer abundance (*m*_*i*_) for each metabolite incorporating *i*^13^C atoms was calculated as follows:

$${m}_{i}( \% )=\frac{{M}_{i}}{{\sum }_{j=0}^{n}{M}_{j}}\times 100$$

where *M*_*i*_ represents the isotopomer abundance for each metabolite incorporating *i*^13^C atoms. The ^13^C fraction of metabolites possessing *n* carbon atoms was calculated as follows:

$${}^{{\rm{13}}}{\rm{C}}\,{\rm{fraction}}=\sum _{i=1}^{n}\frac{i\times {m}_{i}}{n}$$

### Enzyme assays

After 4 h of cultivation, cells were harvested by centrifugation at 3,500 × g for 5 min at 4 °C, washed twice with sterile water, and suspended in 50 mM Tris-HCl buffer (pH 7.5). The suspended cells were mixed with glass beads (0.6-mm diameter) and disrupted using a Shake Master Neo (Bio Medical Science, Tokyo, Japan). The crude extract, collected after centrifugation at 12,000 × g at 4 °C for 30 min, was used for the enzyme assay.

Unless otherwise noted, enzyme activities were assayed by measuring the oxidation of NADH or the production of NADPH in the reaction mixtures using a U-3010 spectrophotometer (Hitachi, Tokyo, Japan). The composition of each reaction was as follows: for malate dehydrogenase (MDH), 45 mM KPO_4_ buffer (pH 7.4), 0.12 mM NADH, and 0.33 mM oxaloacetate^25^; for malic enzyme (MAE), 100 mM Tris-HCl (pH 7.5), 10 mM MgCl_2_, 0.4 mM NADP^+^, and 10 mM L-malate^26^; for pyruvate carboxylase (PYC), 100 mM Tris acetate (pH 8.5), 10 mM KHCO_3_, 10 mM magnesium acetate, 0.1 mM NADH, 2 mM pyruvate, and 1.5 U/ml malate dehydrogenase (Oriental Yeast Co., Ltd., Tokyo, Japan)^27^; and for hexokinase (HXK), 50 mM Tris-HCl (pH 7.5), 6.7 mM MgCl_2_, 1 mM ATP, 0.8 mM NADP^+^, 1 mM glucose, and 1 U/ml glucose-6-phosphate dehydrogenase^28^. Protein concentrations were determined by the Quick Start™ Bradford Protein Assay (Bio-Rad) with bovine serum albumin as the standard^29^. The activities are presented as the mean of at least three independent technical replicates. One unit of enzyme activity was defined as the amount of enzyme that produced 1 µmol of product per min.

## Results

### Time-course profiles of ethanol and acetate by *S*. *cerevisiae* and *K*. *marxianus*

**Figure 1:**
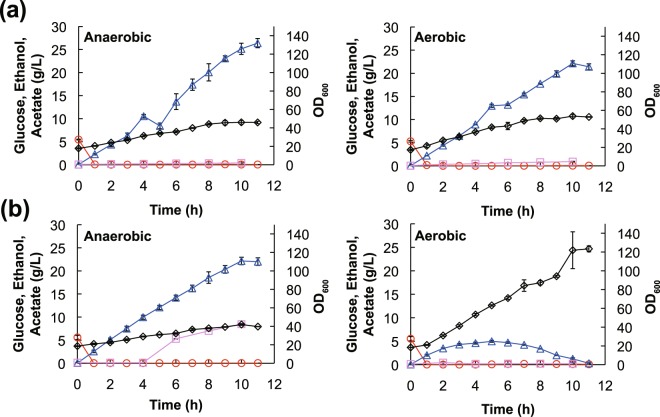
Growth characteristics and metabolite profiles of *S*. *cerevisiae* and *K*. *marxianus*. *S*. *cerevisiae* (**a**) and *K*. *marxianus* (**b**) were cultivated under aerobic (right) or anaerobic (left) conditions. Time-dependent glucose consumption and extracellular ethanol and acetate concentrations were monitored. The symbols used in the figure are as follows: ○, glucose; △, ethanol; ▢, acetate; and ◇, OD_600nm_. Values represent the mean (±SD) of three biological replicates.

*S*. *cerevisiae* and *K*. *marxianus* strains were cultivated (separately) at 30 °C in YP medium containing glucose under aerobic and anaerobic conditions to investigate the effect of aeration on ethanol and acetate production. The initial density of yeast cells was 12.5 g of wet cells/L (OD_600_ = 18). Glucose was continuously fed into the medium at a rate of 5 g/h through 9 h of cultivation, yielding a total addition of 50 g glucose in each 100-mL culture. Ethanol and acetate concentrations in the supernatant were determined by HPLC. The growth of *S*. *cerevisiae* reached the highest cell densities (OD_600_ = 46 and 54) after 10 h of cultivation under anaerobic and aerobic conditions, respectively (Fig. 1a). Under anaerobic conditions, ethanol concentrations increased during cultivation. The highest titer of ethanol was 26.4 g/L after 11 h of cultivation (Fig. 1a). The productivity of ethanol under aerobic conditions was lower than that under anaerobic conditions; the highest titer of ethanol was 22.1 g/L after 10 h of aerobic cultivation. In contrast, *S*. *cerevisiae* gradually accumulated a small amount of acetate (1.3 g/L) under aerobic conditions, while the acetate titer under anaerobic conditions peaked at less than 0.5 g/L. *K*. *marxianus* achieved the highest cell density (OD_600_ = 42) under anaerobic conditions after 10 h of cultivation, while the highest cell density (OD_600_ = 121, 10 h cultivation) under aerobic conditions was almost 3-fold that under anaerobic conditions (Fig. 1b). As in the case of *S*. *cerevisiae* grown under anaerobic conditions, ethanol production by *K*. *marxianus* increased during anaerobic cultivation, peaking at an ethanol titer of 22.1 g/L. However, the ethanol concentration in the *K*. *marxianus* culture increased gradually during the first 5 h of cultivation, with the titer peaking at 0.5 g/L under aerobic conditions, before subsequently decreasing to 0.3 g/L after 11 h of cultivation. Acetate productivity by *K*. *marxianus* increased after 4 and 6 h of cultivation under anaerobic conditions; the highest titer (7.0 g/L) was achieved after 10 h of cultivation (Fig. 1b). On the other hand, the acetate titer peaked at less than 0.5 g/L under aerobic conditions.

### Metabolome analysis

**Figure 2:**
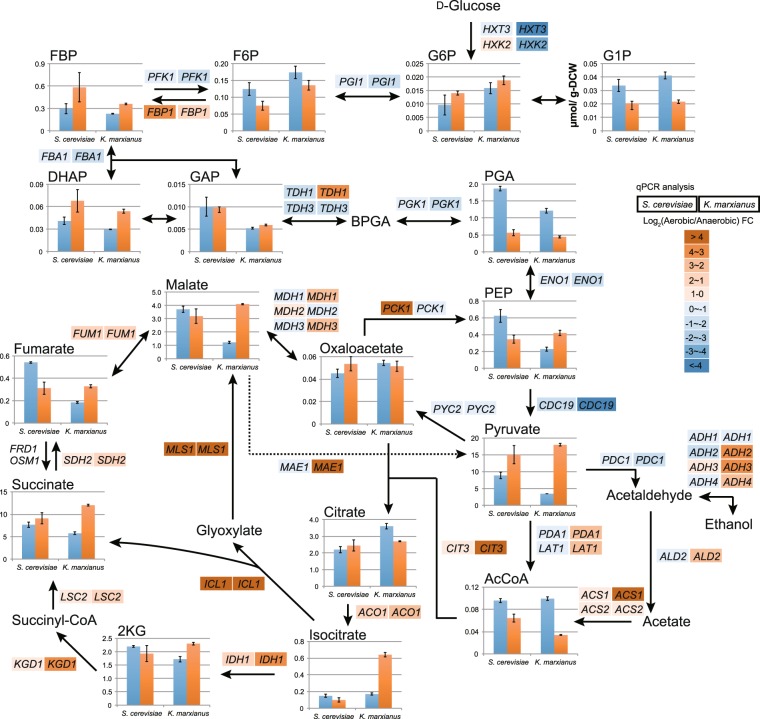
Pool sizes of intracellular metabolites in *S*. *cerevisiae* and *K*. *marxianus*. Each strain was cultivated for 4 h under aerobic (blue) or anaerobic (orange) conditions. Values represent the mean (±SD) of three biological replicates. Changes in transcript abundance are shown, on a log_2_-fold scale, for the genes involved in the indicated pathways. y-axis, µmol/g-DCW.

Intracellular metabolites were extracted from *S*. *cerevisiae* or *K*. *marxianus* cells growing aerobically or anaerobically after 4 h of cultivation. The pool sizes of hexoses (glucose-6-phosphate, G6P; fructose-6-phosphate, F6P; and fructose-1,6-bisphosphate, FBP), triose phosphates (dihydroxyacetone phosphate, DHAP; glyceraldehyde 3-phosphate, GAP; 3-phosphoglycerate, PGA; phosphoenolpyruvate, PEP), organic acids (citrate, isocitrate, 2-ketoglutarate, succinate, fumarate, malate, and oxaloacetate), pyruvate, and acetyl-CoA (AcCoA) in central metabolism were calculated by LC-QqQ-MS analysis based on the areas of the peaks (Fig. 2). In *S*. *cerevisiae* cells, the pool sizes of several metabolites under aerobic conditions were higher than those under anaerobic conditions. These metabolites with elevated pool sizes were as follows: F6P, 1.7-fold; PGA, 3.3-fold; PEP, 1.8-fold; AcCoA, 1.5-fold; isocitrate, 1.5-fold; and fumarate, 1.7-fold (on a log_2_ scale). The combined pool size of FBP, G6P, DHAP, and pyruvate in aerobic cultivation was more than one-half that in anaerobic cultivation. The pool sizes of the other metabolites, including G6P, GAP, citrate, 2-ketoglutarate, succinate, malate, and oxaloacetate, were comparable between aerobic and anaerobic cultivation. In *K*. *marxianus* cells, the changes in metabolite pool size from G6P to PGA between aerobic and anaerobic cultivation were similar to those in *S*. *cerevisiae*. However, unlike the case in *S*. *cerevisiae*, the pool sizes of several metabolites under anaerobic conditions were increased compared to those observed under aerobic conditions, including the following: PEP, 1.8-fold; isocitrate, 3.8-fold; 2-ketoglutarate, 1.3-fold; succinate, 2.1-fold; malate, 3.3-fold; and fumarate, 1.8-fold. Notably, in *K*. *marxianus*, the pool size of pyruvate in anaerobic cultivation was 4.5-fold higher than that in aerobic cultivation (Fig. 2).

### Quantification of transcript abundance by qPCR

Total RNA was extracted from the cells after 4 h of cultivation under aerobic or anaerobic conditions. qPCR was conducted to analyze the mRNA expression levels of individual genes involved in glycolysis and the TCA cycle. Changes in transcript abundance under aerobic and anaerobic conditions are shown, on a log_2_-fold scale, for each gene (Table 1, Fig. 2). In *S*. *cerevisiae*, the levels of transcripts of genes involved in glycolysis (*PGI1*, phosphoglucose isomerase; *PFK1*, phosphofructokinase; *FBA1*, fructose 1,6-bisphosphate aldolase; *PGK1*, 3-phosphoglycerate kinase; *CDC19*, pyruvate kinase; *ENO1*, PEP hydratase) all were decreased under anaerobic conditions. In contrast, the expression level of *HXK2*, which encodes hexokinase, under aerobic conditions was quantitatively (0.55-fold) higher than that under anaerobic conditions. The transcript abundances of three alcohol dehydrogenase-encoding genes (*ADH1*, *ADH2*, and *ADH4*) under aerobic conditions were 0.59-fold, 1.85-fold and 0.79-fold lower than those under anaerobic conditions, respectively, whereas the *ADH3* gene transcript was quantitatively increased (0.6-fold) under aerobic conditions. The *PDA1* and *LAT1* genes, both of which encode pyruvate dehydrogenase subunits, also exhibited decreased transcript levels under aerobic conditions. The transcript abundance of the *MDH1* gene, which encodes mitochondrial malate dehydrogenase, under aerobic conditions was 0.6-fold lower than that obtained under anaerobic conditions. Interestingly, transcripts of *MDH2*, which encodes cytosolic MDH, accumulated to higher levels under aerobic conditions compared to those under anaerobic conditions. In the anaplerotic pathway, the mRNA expression level of the *MAE1* gene, which encodes malic enzyme, under aerobic conditions was less than one-half that under anaerobic conditions, while transcripts of the *S*. *cerevisiae PCK1* gene, which encodes PEP carboxykinase, accumulated to 5.42-fold higher levels under anaerobic conditions than under aerobic conditions.

**Table 1:** Changes in transcript abundance of the genes involved in glycolysis, ethanol metabolism, TCA cycle, and anaplerotic pathway under aerobic and anaerobic conditions.

Gene	Product	Log_2_ FC ± SD* in *S*. *cerevisiae*	Log_2_ FC ± SD^*^ in *K*. *marxianus*
**Glycolysis**			
*HXT3*	Hexose transporter	−0.90 ± 0.35	−4.22 ± 0.65
*HXK2*	Hexokinase II	0.55 ± 0.24	−3.48 ± 0.93
*PGI1*	Glucose-6-phosphate isomerase	−0.56 ± 0.30	−1.31 ± 0.12
*PFK1*	Phosphofructokinase alpha subunit	−0.74 ± 0.49	−1.77 ± 0.33
*FBP1*	Fructose-1,6-bisphosphatase	3.76 ± 0.06	1.83 ± 0.15
*FBA1*	Aldolase	−0.70 ± 0.23	−2.59 ± 0.76
*TDH1*	Glyceraldehyde-3-phosphate dehydrogenase 1	−1.18 ± 0.34	3.86 ± 0.25
*TDH3*	Glyceraldehyde-3-phosphate dehydrogenase 3	−1.23 ± 0.36	−1.73 ± 0.40
*PGK1*	3-Phosphoglycerate kinase	−0.23 ± 0.34	−2.08 ± 1.05
*ENO1*	Enolase 1	−0.90 ± 0.19	−2.18 ± 0.60
*CDC19*	Pyruvate kinase 1	−1.70 ± 0.16	−4.18 ± 1.23
*PDA1*	Pyruvate dehydrogenase complex component E1 alpha	−0.22 ± 0.15	1.92 ± 0.21
*LAT1*	Pyruvate dehydrogenase complex component E2	−0.54 ± 0.49	1.72 ± 0.13
**Ethanol metabolism**			
*PDC1*	Pyruvate decarboxylase isozyme 1	−0.16 ± 0.18	−2.28 ± 0.48
*ADH1*	Alcohol dehydrogenase I, cytoplasmic	−0.59 ± 0.25	−0.01 ± 0.19
*ADH2*	Alcohol dehydrogenase II, cytoplasmic	−1.85 ± 0.34	2.23 ± 0.31
*ADH3*	Alcohol dehydrogenase III, mitochondorial	0.63 ± 0.12	2.63 ± 0.22
*ADH4*	Alcohol dehydrogenase IV, cytoplasmic	−0.79 ± 0.16	1.68 ± 0.30
*ALD2*	Aldehyde dehydrogenase	−0.77 ± 0.43	1.07 ± 0.26
*ACS1*	Acetyl-coenzyme A synthetase 1	0.64 ± 0.30	4.19 ± 0.28
*ACS2*	Acetyl-coenzyme A synthetase 2	0.65 ± 0.10	0.95 ± 0.13
**TCA cycle**			
*CIT3*	Citrate synthase 3, mitochondrial	0.44 ± 0.32	9.99 ± 0.88
*ACO1*	Aconitase, cytoplasmic	1.43 ± 0.30	2.59 ± 0.33
*IDH1*	Isocitrate dehydrogenase, cytoplasmic	1.99 ± 0.09	3.69 ± 0.49
*KGD1*	2-Oxoglutarate dehydrogenase, mitochondrial	0.87 ± 0.28	3.64 ± 0.65
*LSC2*	Succinate-CoA ligase [ADP-forming] subunit beta, mitochondorial	0.21 ± 0.18	2.21 ± 0.39
*SDH2*	Succinate dehydrogenase [ubiquinone] iron-sulfur subunit, mitochondorial	0.43 ± 0.26	1.85 ± 0.22
*FUM1*	Fumarate hydratase, mitochondorial	1.16 ± 0.25	1.73 ± 0.21
*MDH1*	Malate dehydrogenase, mitochondorial	−0.61 ± 0.04	1.74 ± 0.37
*MDH2*	Malate dehydrogenase, cytoplasmic	0.76 ± 0.28	−0.63 ± 0.09
*MDH3*	Malate dehydrogenase, peroxisomal	−0.42 ± 0.49	1.92 ± 0.21
*ICL1*	Isocitrate lyase	4.51 ± 0.19	11.91 ± 0.44
*MLS1*	Malate synthase 1, glyoxysomal	6.00 ± 0.09	5.20 ± 0.33
**Anaplerotic pathway**			
*PCK1*	Phosphoenolpyruvate carboxykinase (ATP)	5.42 ± 0.24	−0.89 ± 1.39
*PYC2*	Pyruvate carboxylase 2	−0.08 ± 0.28	−0.19 ± 0.73
*MAE1*	NAD-dependent malic enzyme, mitochondrial	−0.12 ± 0.22	5.40 ± 0.36

*The Log_2_ FC (Aerobic/anaerobic) is expressed as a log_2_ value with standard deviation (SD).

As in the case of *S*. *cerevisiae*, the transcript abundances of the genes involved in glycolysis were lower under aerobic conditions than under anaerobic conditions in *K*. *marxianus* (Table 1). The decreased abundances of these genes were as follows: *PGI1*, 1.31-fold; *PFK1*, 1.77-fold; *FBA1*, 2.59-fold; *PGK1*, 2.08-fold; *CDC19*, 4.18-fold; and *ENO1*, 2.18-fold (on a log_2_ scale). Decreased transcript abundance under anaerobic conditions also was observed for the *TDH1* gene, which encodes glyceraldehyde 3-phosphate. The *HXK2* gene transcript under aerobic cultivation was 3.48-fold lower than that under anaerobic conditions. The mRNA expression levels of the genes involved in ethanol assimilation or production were increased under aerobic conditions, including the following genes: *ADH2*, 2.23-fold; *ADH4*, 1.68-fold; *ALD2*, 1.07-fold; *ACS1*, 4.19-fold; *PDA1*, 1.92-fold; *LAT1*, 1.72-fold; *MDH1*, 1.74-fold; and *MDH3*, 1.92-fold (on a log_2_ scale). In contrast to the gene expression profile in *S*. *cerevisiae*, under aerobic conditions the transcript of the *K*. *marxianus MAE1* gene accumulated to a level approximately 5.40-fold higher than that under anaerobic fermentation, while the transcript of the *K*. *marxianus PCK1* gene was decreased under aerobic conditions. Note that aeration caused more than 3-fold (on a log_2_ scale) increases in the transcript abundances of the *CIT3*, *IDH1*, and *KGD1* genes, which encode citrate synthase, isocitrate dehydrogenase, and 2-ketoglutrate dehydrogenase, respectively.

### ^13^C-metabolic turnover analysis

**Figure 3:**
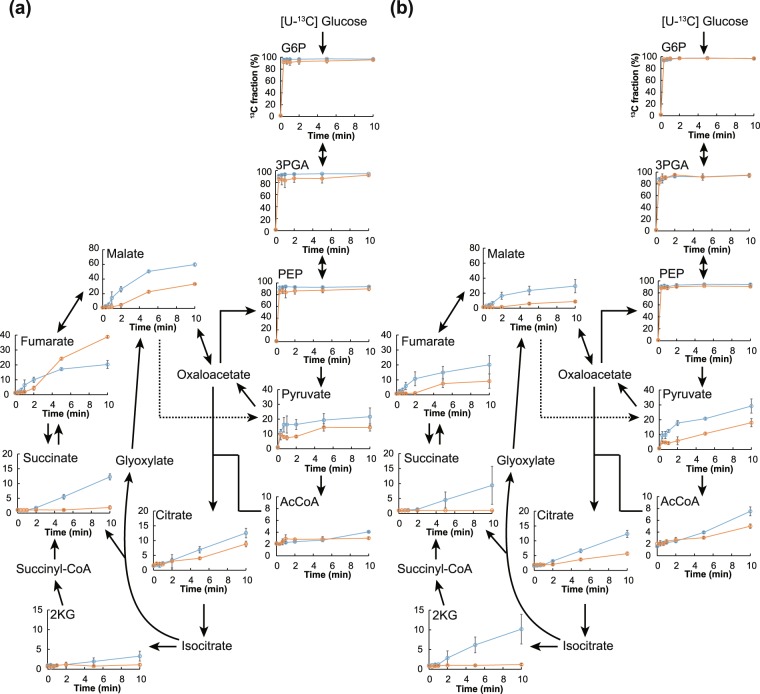
Time-course changes in ^13^C fractions of metabolites in *S*. *cerevisiae* and *K*. *marxianus*. [U-^13^C] Glucose was added to each *S*. *cerevisiae* (**a**) or *K*. *marxianus* (**b**) growth medium after 4 h cultivation under aerobic (blue) or anaerobic (orange) conditions. Values represent the mean (±SD) of three biological replicates. y-axis, ^13^C fraction (%) calculated by equations described in “^13^C-labeling metabolomics” in Methods.

*In-vivo* ^13^C-labeling was performed to analyze the turnover of metabolites in *S*. *cerevisiae* and *K*. *marxianus*. ^13^C was incorporated into metabolites in the yeast cells following the addition of [U-^13^C] glucose after 4 h of cultivation in YPD medium. The ^13^C fraction, defined as the ratio of ^13^C to the total carbon in each metabolite, was calculated from the mass isotopomer distributions. The ^13^C fraction of glycolysis metabolites (G6P, 3PGA, and PEP) reached a maximum of more than 90% after 10 sec of labeling in both yeast species (Fig. 3). On the other hand, the ^13^C incorporation of metabolites via the TCA cycle rose more slowly over time compared to those generated via glycolysis. The ^13^C fractions of TCA cycle metabolites were lower than those of their common precursor, PEP. Aeration promoted ^13^C incorporation into metabolites of the TCA cycle in both yeast species, implying the activation of metabolic turnover via the TCA cycle under aerobic conditions. The ^13^C fraction of fumarate under aerobic conditions was lower than that under anaerobic conditions in *S*. *cerevisiae* after 10 min of labeling. Furthermore, the ^13^C fraction of malate in *S*. *cerevisiae* reached a maximum of about 40% and 60% after 10 min of labeling under anaerobic and aerobic conditions, respectively. The ^13^C labeling of 2-ketoglutarate was about 1% and 3% in *S*. *cerevisiae* under anaerobic and aerobic conditions, respectively. In contrast to the cases in *S*. *cerevisiae*, the ^13^C fraction of malate was around 10% after 10 min of labeling in *K*. *marxianus* grown under anaerobic conditions; however, the corresponding value was 30% under aerobic conditions. The ^13^C labeling of 2-ketoglutarate was less than 1% after 10 min of labeling in *K*. *marxianus* grown under anaerobic conditions, but the corresponding value was approximately 10% after 10 min of labeling under aerobic conditions. These results suggested that (in contrast to *S*. *cerevisiae*), *K*. *marxianus* enhances metabolic turnover in the TCA cycle during aeration.

### Enzyme activity of HXK, MAE, and MDH

**Figure 4:**
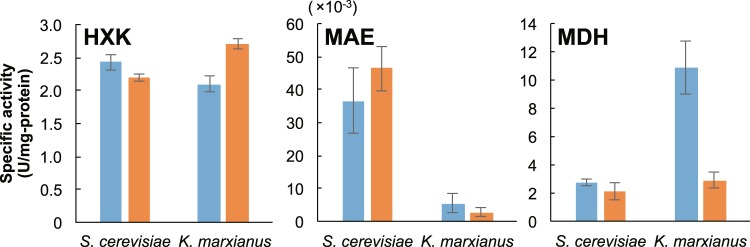
Enzyme activity of *S*. *cerevisiae* and *K*. *marxianus* cell extracts. The specific activities of hexokinase (HXK), malic enzyme (MAE), and malate dehydrogenase (MDH) were determined in crude extracts of *S*. *cerevisiae* and *K*. *marxianus* strains cultivated for 4 h under aerobic (blue) or anaerobic (orange) conditions. Values represent the mean (±SD) of three independent measurements.

*S*. *cerevisiae* or *K*. *marxianus* cells were cultivated for 4 h under aerobic or anaerobic conditions, and cell extracts were obtained by shaking with glass beads. Given the observed difference in the ^13^C-enrichment pattern of malate in the two species, the activities of MDH and MAE, which are responsible for malate metabolism, were measured (Fig. 4). In *S*. *cerevisiae*, MDH and MAE activities were similar in the cells grown under aerobic and anaerobic conditions, while the activity of HXK, the first enzyme of glycolysis, was significantly elevated in cells grown under aerobic conditions compared to the activity in cells grown under anaerobic conditions. This observation was consistent with the earlier observation that the *HXK2* and *HXK3* transcript abundances were decreased under aerobic conditions. The MAE activity of *K*. *marxianus* cells under aerobic conditions was about 2.0–fold higher than that under anaerobic conditions, even though *K*. *marxianus MAE1* transcript was significantly higher than (approximately 5.40-fold) that under anaerobic fermentation. Note that MDH activity was significantly enhanced in *K*. *marxianus* under aerobic conditions when compared to that under anaerobic condition. On the other hand, HXK activity in *K*. *marxianus* cells grown under aerobic conditions was decreased (0.8-fold) compared to that in cells grown under anaerobic conditions.

## Discussion

Here, we have determined the metabolic fluxes of glycolysis and the TCA cycle, as well as transcript abundances of the corresponding genes, in response to aeration in two yeast species, *S*. *cerevisiae* and *K*. *marxianus*. This evaluation is expected to facilitate a comprehensive comparison and understanding of metabolic flux changes in Crabtree-positive and –negative yeast strains. *S*. *cerevisiae* produces ethanol under aerobic conditions when glucose is in excess, generally referred to as the Crabtree effect. The fermentation data obtained in the present work showed that the titer of ethanol was comparable in *S*. *cerevisiae* cultures grown under aerobic and anaerobic conditions (Fig. 1). Among the transcripts of the four *ADH* paralogs, that of *ADH3* accumulated to increased levels under aerobic conditions (Fig. 2). ADH3, which localizes within the mitochondrial fraction, is essential for aerobic growth and controls the intracellular NADH/NAD balance via the supply of NADH^30^. This result implies that ADH3 positively contributes to the metabolic regulatory system, facilitating the Crabtree effect. We hypothesize that the resulting NADH abundance in mitochondria contributes to the activation of pyruvate dehydrogenase kinase, thereby inactivating pyruvate dehydrogenase and favoring the reductive TCA cycle via the reduction by malate dehydrogenase, which converts oxaloacetate to malate^30^. In fact, ^13^C-metabolic turnover analysis yielded a ^13^C fraction of malate of 60% (the maximum observed value) under aerobic conditions (Fig. 4a). In addition, ethanol metabolism is expected to be suppressed under such conditions, given that the transcript abundances of *ADH2* and *ALD2*, both of which are involved in acetate production, were decreased during aerobic growth. Furthermore, *PDA1* and *LAT1* transcript levels decreased under aerobic conditions; this observation may indicate the accumulation of pyruvate, leading to the high production of ethanol via the action of ADH3. Succinate is formed by the reductive TCA cycle during fermentation^31^. Our ^13^C-metabolic turnover analysis revealed that the ^13^C-labeling rate of fumarate under anaerobic conditions was lower than that under aerobic conditions (Fig. 3). The transcript abundances of *S*. *cerevisiae MAE1*, *MDH1*, and *MDH3* were comparable between aerobic and anaerobic conditions, consistent with the observation that the ^13^C fraction of malate peaked at 40%, even under anaerobic conditions.

In contrast to *S*. *cerevisiae*, *K*. *marxianus* produces lower amounts of ethanol and acetate during aerobic growth in the presence of high concentrations of glucose^32^. The ethanol titer obtained during aerobic cultivation of *K*. *marxianus* peaked at 5 g/L before gradually decreasing after 5 h of cultivation; the acetate titer peaked at less than 0.2 g/L during anaerobic cultivation (Fig. 1). This pattern might be explained by the mRNA expression profile of this yeast; that is, the mRNA expression levels of *K*. *marxianus ADH2*, *ALD2*, and *ACS1*, which are responsible for ethanol assimilation to acetyl-CoA, were increased by aeration (Fig. 2). Aerobic growth also yielded increases in the transcript abundances of *PDA1* and *LAT1*; these genes encode activities that contribute to the augmentation of acetyl-CoA synthesis, resulting in a substantial decrease in the pool size of pyruvate (Fig. 2). The increased pool sizes of isocitrate, succinate, fumarate, and malate in *K*. *marxianus* grown under anaerobic cultivation can be attributed to the retardation of metabolic flux via the TCA cycle (Fig. 2). On the other hand, the present ^13^C-labeling data indicated that aeration led to elevation of the ^13^C fraction of TCA cycle metabolites. Together, our ^13^C-metabolic turnover analysis, qPCR, and enzyme assay data revealed that the flux of malate synthesis was increased under aerobic conditions, mainly due to the increase of the expression level of *MDH*. Furthermore, the increased mRNA expression level of *ADH3* was expected to enhance the mitochondrial supply of NADH. In addition, accumulation of the transcripts of the *CIT1*, *IDH1*, and *KGD1* genes, which are involved in the TCA cycle, implied activation of the oxidative TCA cycle. This inference was supported by our ^13^C-labeling data, which revealed that the ^13^C fraction of 2-ketoglutarate was more than 2.7-fold higher during aerobic cultivation compared to anaerobic cultivation (Fig. 3).

A Crabtree-negative strain of *S*. *cerevisiae* with a faster growth rate recently was developed by modification of a pyruvate decarboxylase-deficient strain via engineering of pyruvate metabolism and adaptive laboratory evolution^9^. Glucose uptake was restricted in the engineered *S*. *cerevisiae* strain to avoid unrestricted glucose uptake, therefore, this modification limited the strain’s capacity for reoxidation of cytosolic NADH. Separate work has shown that abolishment of pyruvate kinase PYK1 activity results in decreased glycolytic flux, relieving the Crabtree effect^33^. Moreover, the multi-copy presence of hexose transporter-encoding genes has been shown to enhance ethanol production by fermentation under aerobic conditions^34^. In the present study, we showed that glucose uptake is restricted in *K*. *marxianus* under aerobic conditions, correlating with decreases in hexokinase at the level of both mRNA expression and enzymatic activity (Figs 3 and 4). This finding indicates that controlling glucose uptake is a common strategy to retain efficient carbon and electron flux in the cell, enabling the maintenance of high growth rate. We conclude that *K*. *marxianus* growing under aerobic conditions augments acetyl-CoA biosynthesis from pyruvate and employs the oxidative TCA cycle; in combination with a decrease in the glucose uptake rate, these effects lead to strong suppression of ethanol and acetate production. The data presented here are expected to serve as a promising platform for the rational design of *K*. *marxianus* strains appropriate for the production of bio-based chemicals, as part of a metabolic engineering strategy.

## Supplementary information

Supplemental Table 1
