# Supplementary material for: Increased flux in acetyl-CoA synthetic pathway and TCA cycle of Kluyveromyces marxianus under respiratory conditions
Source: Sci Rep. 2019 Mar 29;9:5319. doi: 10.1038/s41598-019-41863-1 (PMC6440987; doi:10.1038/s41598-019-41863-1)
Supplement: Supplementary file 1 — Supplemental Table 1 [file 41598_2019_41863_MOESM1_ESM.docx]

**Supplementary Information**

**Increased flux in acetyl-CoA synthetic pathway and TCA cycle of *Kluyveromyces marxianus* under respiratory conditions**

Yuri Sakihama^1^, Ryota Hidese^1^, Tomohisa Hasunuma^1^*, Akihiko Kondo^1,2^*

^1^Graduate School of Innovation, Science and Technology, Kobe University, 1-1 Rokkodai, Nada, Kobe 657-8501, Japan. ^2^RIKEN Center for Sustainable Resource Science, 1-7-22 Suehiro, Tsurumi, Yokohama, Kanagawa 230-0045, Japan.

*Tomohisa Hasunuma, hasunuma@port.kobe-u.ac.jp

Supplemental Table 1. Primers used in this study.

| Gene | *K. marxianus* |  |  | *S. cerevisiae* |  |
| --- | --- | --- | --- | --- | --- |
|  | Primers | Sequence |  | Primers | Sequence |
| *HXT3* | RT-PCR_hxt3_F | TGTCTTTTGCCTGGGCCATT |  | Sc.RT-PCR_HXT3_F | ATTCGAATGCTGACCTGCCT |
|  | RT-PCR_hxt3_R | ATCATCGGTTGCGACCTTGT |  | Sc.RT-PCR_HXT3_R | AACCGAAAACGAAACCACCG |
| *HXK2* | RT-PCR_hxk2_F | CCCAGCCTCCCAAAACAAGA |  | Sc.RT-PCR_HXK2_F | TTGCGTTTGGCCTTGATGGA |
|  | RT-PCR_hxk2_R | GTGGCCTTCGACACCTTCAA |  | Sc.RT-PCR_HXK2_R | TCCTCGATTCTGGCTGGGTA |
| *PGI1* | RT-PCR_pgi1_F | CCCTTGAACATGGCGTCTCT |  | Sc.RT-PCR_pgi1_F | TTGACTCCCACAGTGATGGC |
|  | RT-PCR_pgi1_R | TAAGGACAGCTCTCGTTTCG |  | Sc.RT-PCR_pgi1_R | TGAAATGCCACCGGGTAGAC |
| *PFK1* | RT-PCR_pfk1_F | GTACCACCTTCGCATGACCA |  | Sc.RT-PCR_PFK1_F | CATCGAAGACAACCAAGCTGC |
|  | RT-PCR_pfk1_R | GCCATTTTCAAGGGCTGCAA |  | Sc.RT-PCR_PFK1_R | GCCAGTGAATGACCTTTGGC |
| *FBP1* | RT-PCR_fbp1-2_F | TTGCGTTCAAGTTCATATCACAGAC |  | Sc.RT-PCR_FBP1_F | TCCTAACTTAAGAATTCCGCCTCAA |
|  | RT-PCR_fbp1-2_R | CGCATTAATGAATATCTCGTCACCC |  | Sc.RT-PCR_FBP1_R | TTATAGTCTCGTTCCAGTAGAGGGT |
| *FBA1* | RT-PCR_fba1_F | GGGCCAATGGTTGGAAATGG |  | Sc.RT-PCR_FBA1_F | AACCTCTAACGGTGGTGCTG |
|  | RT-PCR_fba1_R | GGCCTTGTAAACACCGTGGA |  | Sc.RT-PCR_FBA1_R | ATCTGATGTAGTGGGCAGCG |
| *TDH1* | RT-PCR_TDH1_F | AGAGCCTTCACCAAAGGAGC |  | Sc.RT-PCR_TDH1_F | GTCCATCCCACAAGGACTGG |
|  | RT-PCR_TDH1_R | CCATCCAAGACTGCCCCAAT |  | Sc.RT-PCR_TDH1_R | CGACGGTTGGGACTCTGAAA |
| *TDH3* | RT-PCR_TDH3_F | GTCGACGGCAATGTCAACAC |  | Sc.RT-PCR_TDH3_F | GTCCATCCCACAAGGACTGG |
|  | RT-PCR_TDH3_R | TGAAGCAGCACATGGAGCAA |  | Sc.RT-PCR_TDH3_R | CGACGGTTGGGACTCTGAAA |
| *PGK1* | RT-PCR_pgk1_F | GGTCCAGAGACCAGAAAGGC |  | Sc.RT-PCR_PGK1_F | GCTGCTTTGCCAACCATCAA |
|  | RT-PCR_pgk1_R | GACGGCGTCCAATGAGTGTA |  | Sc.RT-PCR_PGK1_R | GGCTTCAACTTCTGGACCGA |
| *Eno1* | RT-PCR_Eno1_F | TGGTTCGGTCTTAGCCTTGG |  | Sc.RT-PCR_ENO1_F | TCCAAGTGGTTGACTGGTCC |
|  | RT-PCR_Eno1_R | ATCTTGGGTGTCTCTTTGGCT |  | Sc.RT-PCR_ENO1_R | GTGAGACCAAGCTTCCCAGT |
| *CDC19* | RT-PCR_cdc19_F | ACCCTTAGCGGTTTCACCAG |  | Sc.RT-PCR_CDC19_F | TTGCTTTGGACACCAAGGGT |
|  | RT-PCR_cdc19_R | TAACTTGGCCGGTAAGCCAG |  | Sc.RT-PCR_CDC19_R | AGCGTACTTGTCATCGGTGG |
| *PDC1* | RT-PCR_pdc1_F | AGCATCCATGACTGGCAACA |  | Sc.RT-PCR_PDC1_F | TTCCAGACACGACGTCAAGG |
|  | RT-PCR_pdc1_R | GGACTACGAAGCCGTCAGAG |  | Sc.RT-PCR_PDC1_R | CCGACGTAAACACCACCGTA |
| *ADH1* | RT-PCR_adh1_F | GGTGACCTTGCCGGTATCAA |  | Sc.RT-PCR_ADH1_F | CAACTGTCCTCACGCTGACT |
|  | RT-PCR_adh1_R | AGAACCGTCGTGGGTGTAAC |  | Sc.RT-PCR_ADH1_R | ACCTTGAGGAATGTGAGCGG |
| *ADH2* | RT-PCR_adh2_F | GTTGGTTCTTACGTTGGTAACAGAG |  | Sc.RT-PCR_ADH2_F | AGAATACGCTACCGCTGACG |
|  | RT-PCR_adh2_R | AATTCTACCAATGATTTGGCCCTTG |  | Sc.RT-PCR_ADH2_R | CCTGCTCTCAAGTTGGCAGA |
| *ADH3* | RT-PCR_adh3_F | GCTGTGCAAGCTGCAAGAAT |  | Sc.RT-PCR_ADH3_F | AATCCACAGCTGCAATCCCT |
|  | RT-PCR_adh3_R | GCCAAGGAACCTAGACCACC |  | Sc.RT-PCR_ADH3_R | ATTTGGCTTAGGCTCGGGG |
| *ADH4* | RT-PCR_adh4_F | GAGATCGGTGACTACGCAGG |  | Sc.RT-PCR_ADH4_F | GGTTTGCCACCTGCTTTGAC |
|  | RT-PCR_adh4_R | GCGTCAGCAGTGGCATATTG |  | Sc.RT-PCR_ADH4_R | AAAGCACAGGCATCGGTGAT |
| *ALD2* | RT-PCR_ALD2_F | TTATGGTGCCATCGCAGGTT |  | Sc.RT-PCR_ALD2_F | TGATGACGCTCTGAAGCTGG |
|  | RT-PCR_ALD2_R | CGGCAAAAATGACGCAAGGA |  | Sc.RT-PCR_ALD2_R | CCGCCAAAAGGAACGGTAAC |
| *ACS1* | RT-PCR_acs1_F | ACCATTGGTGAACCAGGCAT |  | Sc.RT-PCR_ACS1_F | ATCCATGCACACCCGTTGAT |
|  | RT-PCR_acs1_R | CGGGCTCAGCTGTTGAATTG |  | Sc.RT-PCR_ACS1_R | AGTGTGGCCTGTAATCCAGC |
| *ACS2* | RT-PCR_acs2_F | GTGGCGGTACCAAGAGTCAA |  | Sc.RT-PCR_ACS2_F | CAGGGGTAGAGTTGACGACG |
|  | RT-PCR_acs2_R | CTCCACCGCAGGTTACTTGT |  | Sc.RT-PCR_ACS2_R | AGCTTCCGAGACGTTTTCGT |
| *PDA1* | RT-PCR_pda1_F | CTTGTACGCCCCAGGTTTCT |  | Sc.RT-PCR_PDA1_F | TAAGAAGGCCCCTGAGGACA |
|  | RT-PCR_pda1_R | GGAAGCACCATCACCGTACA |  | Sc.RT-PCR_PDA1_R | TACAAGGCGTCACAAGCCAT |
| *LAT1* | RT-PCR_lat1_F | GGTGACCGTTTGTTGCACTC |  | Sc.RT-PCR_LAT1_F | TCACTGTTGCGGCTAAGAGG |
|  | RT-PCR_lat1_R | TCTCTTAGCTGCAACGGCAA |  | Sc.RT-PCR_LAT1_R | GGCACGCTTGACTAGTTCCT |
| *PYC2* | RT-PCR_pyc2_F | GGCACGCAACATCTTACGAC |  | Sc.RT-PCR_PYC2_F | TTGTTATTCCAAGCTCAACAACTGG |
|  | RT-PCR_pyc2_R | TCGCTGGTTCGGTCATTTCA |  | Sc.RT-PCR_PYC2_R | TGAATTGAGCTAAATCACCGACAAC |
| *CIT3* | RT-PCR_cit3_F | CCTGCTCTGGAGTAGGAGGT |  | Sc.RT-PCR_CIT3_F | GCATGGGTTAGCAGCACAAG |
|  | RT-PCR_cit3_R | CTTTGGATCCTGCCGAAGGT |  | Sc.RT-PCR_CIT3_R | ATCGAGGATCCGGTTTACGC |
| *ACO1* | RT-PCR_aco1_F | CGCTTTCGCTATTGCTGGTG |  | Sc.RT-PCR_ACO1_F | GCAGAATGTGGAAACGCTGG |
|  | RT-PCR_aco1_R | AAGAACGGTCCTTTGGTGGG |  | Sc.RT-PCR_ACO1_R | CAGGCAACACGATCTGGTCT |
| *IDH1* | RT-PCR_idh1_F | TTCTGCCTTGATCGGTGGTC |  | Sc.RT-PCR_IDH1_F | AGGCCTGGAACATGAATCCG |
|  | RT-PCR_idh1_R | CTTGGAGATTCTGTCGGCGT |  | Sc.RT-PCR_IDH1_R | TCTTGGCGAAGTCAAAGGCA |
| *KGD1* | RT-PCR_kgd1_F | GGGTTCCGTTACCCCAGAAG |  | Sc.RT-PCR_KGD1_F | TCGTGGGCATACACAGAACC |
|  | RT-PCR_kgd1_R | TACCCAAGACGACAGGGTCT |  | Sc.RT-PCR_KGD1_R | AAAGGCATCTTCTTCGGCCA |
| *LSC2* | RT-PCR_lsc2_F | GACCATTGAGACCGCCTTCA |  | Sc.RT-PCR_LSC2_F | GTCAATGGTGCTGGTTTGGC |
|  | RT-PCR_lsc2_R | CCTTGCGCAACAAAGTCACA |  | Sc.RT-PCR_LSC2_R | TTTGATGGTCTCAGGGGTGG |
| *SDH2* | RT-PCR_sdh2_F | TGAGGCCTCGGGTATCAGAA |  | Sc.RT-PCR_SDH2_F | CCAGACGAGCCAAGTGCTAA |
|  | RT-PCR_sdh2_R | GCAATGGCTCTACCTGGGTT |  | Sc.RT-PCR_SDH2_R | CTAGCGTGTTTCTACCGCCA |
| *FUM1* | RT-PCR_fum1_F | GTGTTGGAACTCGTCGGACT |  | Sc.RT-PCR_FUM1_F | ATGCCACGCCTTTGACACTA |
|  | RT-PCR_fum1_R | TTGGTGGTGAGTTGGGATCG |  | Sc.RT-PCR_FUM1_R | TGTACCAACGGCAGTACCAC |
| *MDH1* | RT-PCR_mdh1_F | GTACCGACCCAACCACTGAG |  | Sc.RT-PCR_MDH1_F | GGTGTTGCCACCGATTTGTC |
|  | RT-PCR_mdh1_R | GTCCAATTCCTCGCCCTTGA |  | Sc.RT-PCR_MDH1_R | ATACCAGGCTTTCTGGGCAC |
| *MDH2* | RT-PCR_mdh2_F | ATACCTGTTAGTGAGCGGAACATAC |  | Sc.RT-PCR_MDH2_F | GTCTCATATAGACACCCCCATTTCC |
|  | RT-PCR_mdh2_R | CTTCAAAATCGCAGCACAATTCATC |  | Sc.RT-PCR_MDH2_R | CCTGCAGGAATGACAACAATAGAAG |
| *MDH3* | RT-PCR_mdh3_F | AGTTGGGCACCTCTGTTGAG |  | Sc.RT-PCR_MDH3_F | GGTCCAGTTCGGAGGTGATG |
|  | RT-PCR_mdh3_R | TTTGTCTGCTGCGAAAAGGC |  | Sc.RT-PCR_MDH3_R | ATGCGGAAAGTGACTCCGTT |
| *ICL1* | RT-PCR_icl1_F | AGTAGTGGTCCCTCGAGTCC |  | Sc.RT-PCR_ICL1_F | TTGATTAGCTCAACCATCGATACCA |
|  | RT-PCR_icl1_R | GCGGGTAGATGTGTCATCCC |  | Sc.RT-PCR_ICL1_R | GTTCAATGTCAGCTAGTTCTTGTCC |
| *MLS1* | RT-PCR_mls1_F | CTGTGCAAAAGCACGACGAA |  | Sc.RT-PCR_MLS1_F | TGGGCAAAGGTCCTTACTTCTATTT |
|  | RT-PCR_mls1_R | GAGGTGCTCGTGGATGTTGA |  | Sc.RT-PCR_MLS1_R | GGGATCCCAATGTAATCTTGAGCTA |
| *MAE1* | RT-PCR_mae1_F | CGGATCTCCATTCCCACCAG |  | Sc.RT-PCR_MAE1_F | TGGTGTACGTATTGCTATCTCCAAA |
|  | RT-PCR_mae1_R | GTCACCAGGCTTTAGAGGCG |  | Sc.RT-PCR_MAE1_R | ACGGGCTAGTTTCTTGTTGTTAGTA |
| *PCK1* | RT-PCR_pck1_F | TTGAGGACCAGCAGCCAAAA |  | Sc.RT-PCR_PCK1_F | CTGGTTACACTTCTAAAATGGCTGG |
|  | RT-PCR_pck1_R | ACTGGGTTGAAGGTGAAGCC |  | Sc.RT-PCR_PCK1_R | AACATGGTTGCGTATCTAATAGGGT |
